# An Efficient Algorithm for Direction Finding against Unknown Mutual Coupling

**DOI:** 10.3390/s141120064

**Published:** 2014-10-24

**Authors:** Weijiang Wang, Shiwei Ren, Yingtao Ding, Haoyu Wang

**Affiliations:** School of Information and Electronics, Beijing Institute of Technology, 5 South Zhongguancun Street, Haidian District, Beijing 100081, China; E-Mails: wangweijiang@bit.edu.cn (W.W.); renshiwei@bit.edu.cn (S.R.); wanghaoyu1@bit.edu.cn (H.W.)

**Keywords:** direction of arrival, mutual coupling, subspace, refinement, iteratively

## Abstract

In this paper, an algorithm of direction finding is proposed in the presence of unknown mutual coupling. The preliminary direction of arrival (DOA) is estimated using the whole array for high resolution. Further refinement can then be conducted by estimating the angularly dependent coefficients (ADCs) with the subspace theory. The mutual coupling coefficients are finally determined by solving the least squares problem with all of the ADCs utilized without discarding any. Simulation results show that the proposed method can achieve better performance at a low signal-to-noise ratio (SNR) with a small-sized array and is more robust, compared with the similar processes employing the initial DOA estimation and further improvement iteratively.

## 1. Introduction

Direction finding of multiple sources has received tremendous attention in the field of radar, sonar, mobile communication, and so on. High resolution algorithms, such as Multiple Signal Classification (MUSIC) [1] and Estimation of Signal Parameters via Rotational Invariance Techniques (ESPRIT) [2], can distinguish closely-spaced sources by the use of subspace theory and, therefore, have been widely used in the past few decades [3]. In spite of the potential advantages of the eigenstructure methods, their direct application to real systems is difficult, due to the critically required precise knowledge of the array manifold. In other words, the performance of the super-resolution techniques is strongly dependent on the array manifold accuracy. In practice, the array manifold is inevitably affected by mutual coupling, position perturbation and array gain or phase uncertainties. This results in significant distortion of the amplitude and phase of the signals received from the array. The direct employment of the eigenstructure-based methods leads to a serious degradation of direction finding [4–7]. Array calibration is an effective way to alleviate the deviation of direction of arrival (DOA) estimation.

Several calibration algorithms have been discussed in the last few decades. The earliest investigation was made by Schmidt [8] and Weiss [9]. They measured and stored the array manifold directly by interpolation. However, a large amount of memory is required, and the size and cost of the system could obviously be increased. In order to overcome the drawbacks of the above scheme, a kind of calibration method was proposed using a set of calibration sources in known locations. The maximum-likelihood approach [10] can be used to estimate the calibration matrix for array error compensation. Similarly, the algorithm in [11] was developed utilizing an iterative least mean squares approach. Although these techniques can obtain high accuracy and a large scope of calibration, it is impractical to set a collection of calibration sources and get their DOAs exactly as prior knowledge.

An approach to mitigate the influence of array errors is to calibrate the array by the use of the received signals. Such methods for estimating the DOA, unknown coupling, gain and phase simultaneously are called self-calibration. Friedlander and Weiss proposed to use an iterative process to acquire the parameters and DOA [12,13]. Svantesson formulized it as an optimization problem and solved the problem iteratively to estimate the mutual coupling coefficients for coupling compensation in the linear array of dipoles [14]. However, the result will converge to the local optimum if the initial values deviate far from the real ones. Alternatively, another kind of method was proposed conducting multidimensional search based on subspace fitting. The maximization of *a posteriori* (MAP) estimator proposed in [15] and [16] is one of these algorithms. Although it does not have the problem of convergence, non-linear multidimensional optimization in these methods is computationally consuming, and the convergence rate is relatively slow.

In recent years, several algorithms have been developed based on the characterization of the mutual coupling matrix (MCM). In [17], Ye obtained the initial estimation of DOA by setting the instrumental sensors on each side of the array. As a result, a one-dimensional search of the spatial spectrum can be performed directly using the original array data. Moreover, the result can be refined iteratively by estimating the mutual coupling coefficients for compensation. In [18], the signals received from the middle subarray were directly exploited for the traditional MUSIC, whereas the MCM is assumed to be a complex symmetric Toeplitz matrix. In order to increase the performance of DOA estimation, another method [19] proposed recently takes advantage of the special structure of MCM to parameterize the steering vector. It achieves the estimation of DOA using the whole array and improves the result by mutual coupling compensation.

In this paper, we assume that the MCM is a band-symmetric Toeplitz matrix with finite non-zero elements. By using the parameterized steering vector in [19], the preliminary DOA estimates can be obtained with the whole array. Further compensation of mutual coupling is then made by estimating the ADC first with the orthogonality of the subspace and the mutual coupling coefficients following with the full information of the ADCs. Simulation results show that the proposed algorithm has satisfactory performance compared with the methods in [17] and [19], especially when the SNR is low and small number of sensors exist.

The remainder of the paper is organized as follows. Section 2 is devoted to the problem formulation. Section 3 addresses the direction finding with initial estimation of DOA and coupling compensation by using the subspace theory and all of the parameters estimated. Section 4 gives the concluding remarks.

## 2. Problem Formulation

We consider a uniform linear array (ULA) consisting of *M* sensors with *K* narrowband far-field sources *s*_1_(*t*), *s*_2_(*t*),…, *s_K_*(*t*) received. The sources come from directions *θ*_1_, *θ*_2_, …, *θ_K_* with respect to the normal line of the array. Assume that the distance between adjacent sensors is *d* and the wavelength of the carrier is λ. With the interactions between sensors, the mutual coupling effect cannot be ignored. The *M* × 1 output vector of the array is then given by:

(1) x(t)=CAs(t)+n(t)

where **x**(*t*) = [*x*_1_(*t*), *x_2_*(*t*), …, *x_M_* (*t*)]^T^, **s**(*t*) = [*s*_1_(*t*), *s_2_*(*t*), …, *s_K_* (*t*)]^T^ and **n**(*t*) = [*n*_1_(*t*), *n*_2_(*t*),…,*n_M_*(*t*)]^T^ denote the received signal vector, source signal vector and noise vector, respectively. The notation [·]^T^ denotes the transposition. **A** = [**a**(*θ*_1_), **a**(*θ*_2_), …, **a**(*θ_K_*)] is the array manifold matrix, in which **a**(*θ_k_*) = [1, *β*(*θ_k_*), *β*^2^(*θ_k_*), …, *β^M^*^−1^(*θ_k_*)]^T^ is the steering vector with *β*(*θ_k_*) = exp{−j2*πd* sin *θ_k_/λ*}. C ∈ ℂ*^M^*^×^*^M^* is the MCM, which is generally considered to be independent of the angle [20]. It indicates the interactions between arbitrary two sensors of the array. Equation (1) is obtained under the assumption that the additive sensor noises *n_i_*(*t*), *i* = 1, …, *M* are independent and identically distributed (i.i.d.) white Gaussian with the common variance *σ*^2^*. s_i_*(*t*) and *n_i_*(*t*) are zero-mean wide-sense stationary random processes.

Several studies of the coupling model [12,21] have shown that the coupling between a pair of sensors is nearly the same. Therefore, the MCM is a banded symmetric Toeplitz matrix for the ULA. Furthermore, based on the fact that the mutual coupling between two sensors is inversely proportional to their distance, the coefficient will be zero if two sensors are several wavelength apart. Let *c_ij_* = *c_ji_* = *c*_|_*_i_*_−_*_j_*_|_ denote the mutual coupling coefficient between the *i*-th and the *j*-th element of the ULA, and assume that there are *P* distinct non-zero elements in the MCM with the self coupling *c*_0_ normalized as one. Then, the *M* × *M* matrix C can be expressed as:

(2) $$\text{C}=\text{Toeplitz}\{1,c_{1},\ldots ,c_{P-1},0_{M-P}\}$$

where Toeplitz{·} denotes a symmetric Toeplitz matrix constructed by the *P* × 1 vector **c** = [1, *c*_1_,…, *c_P_*_−1_]^T^. Define:

(3) $${\text{a}}_{m}(\theta )=\text{Ca}(\theta )$$

as the equivalent steering vector of the direction *θ*. From Equation (1), the covariance matrix of the received signals is:

(4) $${\text{R}}_{x}=\text{E}[\text{x}(t){\text{x}}^{\text{H}}(t)]={\text{CAR}}_{S}{\text{A}}^{\text{H}}{\text{C}}^{\text{H}}+\sigma^{2}\text{I}={\text{A}}_{m}{\text{R}}_{S}{\text{A}}_{m}^{\text{H}}+\sigma^{2}\text{I}$$

where (·)^H^ denotes the Hermitian transpose operation, **R***_s_* = **E**[s(*t*)s^H^(*t*)] is the covariance matrix of s(*t*) and **A***_m_* = [**a***_m_*(*θ*_1_), **a***_m_*(*θ*_2_),…, **a***_m_*(*θ_K_*)] is the equivalent array manifold matrix. As demonstrated in [2], the above equation can be rewritten as:

(5) $${\text{R}}_{x}=[{\text{E}}_{S},{\text{E}}_{n}]\Lambda {\left[{\text{E}}_{S},{\text{E}}_{n}\right]}^{\text{H}}$$

using the eigenvalue decomposition (EVD) of **R***_x_*, where Λ = diag{Λ_s_, Λ_n_} is a matrix with *M* eigenvalues at the diagonal in descending order and zero elsewhere. **E***_s_* ∈) ℂ*^M×K^* and **E***_n_* ∈) ℂ*^M×^*^(^*^M^*^−^*^K^*^)^ are the eigenvectors corresponding to the *K* largest eigenvalues and the *M* − *K* smallest eigenvalues, respectively. According to the well-known subspace-based algorithm [1], the signal subspace spanned by the column of **A***_m_* = **CA** is orthogonal to the noise subspace spanned by that of **E***_n_*. Therefore, we have:

(6) $${\left\|{\text{E}}_{n}^{H}{\text{a}}_{m}(\theta )\right\|}^{2}=0$$

If the MCM is known, the directions of sources *θ*_1_, *θ*_2_, …, *θ_K_* can be estimated based on the spectrum function $P(\theta )=1/{\left\|{\text{E}}_{n}^{H}{\text{a}}_{m}(\theta )\right\|}^{2}$. However, the vector c is usually unknown, under which circumstance the traditional DOA estimation approach cannot be used, and a new method should be investigated for direction finding.

## 3. Direction Finding and Mutual Coupling Compensation

In this section, we first reformulate the equivalent steering vector by parameterizing the MCM. Then, we solve the DOA estimation problem in the presence of unknown mutual coupling by applying the whole array data for better performance [19,22]. For further refinement, a new mutual coupling compensation algorithm is proposed by utilizing the special structure of the reformulated MCM with no information lost.

### 3.1. DOA Estimation Using the Whole Array

Let *r_k_* represents the *k*-th element of the *M* × 1 equivalent steering vector a*_m_*(*θ*). By combining Equations (2) and (3), a*_m_*(*θ*) can be expressed as:

(7) $${\text{a}}_{m}(\theta )={\left[r_{1},r_{2},\ldots ,r_{M}\right]}^{\text{T}}$$

where:

$$\begin{array}{ccc} r_{k}= & [g_{1}+\sum_{i=1}^{k-1}c_{i}\beta^{-i}(\theta )]\beta^{k-1}(\theta ), & k=1,\ldots ,P-1\\ r_{k}= & g_{0}\beta^{k-1}(\theta ), & k=P,\ldots ,M-P+1\\ r_{k}= & [g_{-1}+\sum_{i=1}^{M-k}c_{i}\beta^{i}(\theta )]\beta^{k-1}(\theta ), & k=M-P+2,\ldots ,M \end{array}$$

For notational clarity, we set $\sum_{1}^{0}c_{i}\beta^{\pm i}(\theta )=0$, $g_{1}=\sum_{i=0}^{P-1}c_{i}\beta^{i}(\theta )$, $g_{-1}=\sum_{i=0}^{P-1}c_{i}\beta^{-i}(\theta )$ and $g_{0}=1+\sum_{i=1}^{P-1}c_{i}(\beta^{i}(\theta )+\beta^{-i}(\theta ))$ in the above equations. In the case of *g*_0_ ≠ 0, we can extract *g*_0_ out of *r_k_* and describe a*_m_*(*θ*) as [19]:

(8) $${\text{a}}_{m}(\theta )=g_{0}\Gamma (\theta )\text{a}(\theta )$$

where: **Γ**(*θ*) = *diag* {*μ*_1_, …, *μ_p_*_−1_, 1, …, *α*_1_, …, *α_p_*_−1_} with *M* − 2(*P* − 1) ones,

(9) $$\begin{array}{ll} \mu_{k}=r_{k}\beta^{1-k}(\theta )/g_{0}, & k=1,\ldots ,P-1 \end{array}$$

and:

(10) $$\begin{array}{ll} \alpha_{k}=r_{k+(M-P+1)}\beta^{1-k}(\theta )/g_{0}, & k=1,\ldots ,P-1 \end{array}$$

where *μ_k_* and *α_k_* are the ADCs. They are functions of *C_k_* and *β*(*θ*). It has been shown from Equations (8)–(10) that the angularly independent MCM is transformed into an angularly-dependent expression *g*_0_**Γ**(*θ*). Since **Γ**(*θ*) is a diagonal matrix with 2(*P* − 1) unknown variables and a(*θ*) is a vector, it is feasible to exchange their elements and to reduce the number of ones in **Γ**(*θ*) as follows [19]:

(11) $${\text{a}}_{m}(\theta )=g_{0}\text{T}(\theta )\text{v}(\theta )$$

**v** (*θ*) = [*μ*_1_, …, *μ_P_*_−1_, 1, *α*_1_, …, *α_P_*_−1_]^T^ is an (2*P* − 1) × 1 vector, and:

(12) $$\text{T}\left(\theta \right)=\left[\begin{array}{cccccc} 1 & & & & & \\ & \beta (\theta ) & & & & \\ & & \ddots & & & \\ & & & \beta^{P-1}(\theta ) & & \\ & & & \vdots & & \\ & & & \beta^{M-P}(\theta ) & & \\ & & & & \ddots & \\ & & & & & \beta^{M-1}(\theta ) \end{array}\right]$$

is an *M* × (2*P* − 1) matrix with an (*M* − 2*P* + 2) × 1 vector and two (*P* − 1) × (*P* − 1) diagonal matrices locatedat the diagonal, respectively

Considering the subspace principle given in Equation (6) and a*_m_*(*θ*) in Equation (11), we have the following equation on condition that *g*_0_ ≠ 0:

(13) $${\text{v}}^{\text{H}}(\theta )\text{Q}(\theta )\text{v}(\theta )=0$$

where $\text{Q}(\theta )={\text{T}}^{\text{H}}{\text{E}}_{n}{\text{E}}_{n}^{\text{H}}\text{T}(\theta )$ is a (2*P* − 1) × (2*P* − 1) matrix. As mentioned in [19] and [22], when *θ* is consistent with any one of the *K* incoming angles, **Q**(*θ*) is rank deficient. Therefore, the following spectrum function with the determinant of **Q**(*θ*) as the sensitive factor provides an effective means of DOA estimation:

(14) $$P(\theta )=\frac{1}{\det\{\text{Q}(\theta )\}}$$

It is worth noting that **Q**(*θ*) does not contain any information of **c**. Therefore, the spectrum function given above can be employed even if the mutual coupling is unknown. Compared with the algorithm in [17], this spectrum function takes advantage of the whole array. People do not need to extract the middle array for mutual coupling eliminating. As a result, no information is lost in the spectrum estimation. This method is available only if:

(15) K≤M−2P+1

in which circumstance, the (*M* − *K*) × (2*P* − 1) matrix **E**^H^**T**(*θ*) is full column rank.

### 3.2. Mutual Coupling Compensation

Once we get the DOA estimates, further refinement can be conducted based on the angularly dependent expression of a*_m_*(*θ*) as shown in Equation (11). We assume *θ̂* is the DOA estimated from Equation (14) and that no blind angles exists (*i.e.*, *g*_0_ ≠ 0). Based on the fact that the noise subspace is orthogonal to the column subspace of a*_m_*(*θ̂*), we have:

(16) $${\text{E}}_{n}^{\text{H}}\text{T}(\hat{\theta })\text{v}(\hat{\theta })=0_{(M-K)\times 1}$$

Notice that the *P*-th element of v(*θ̂*) is one, and the others are ADCs to be estimated. Denote $\text{Q}(\hat{\theta })={\text{E}}_{n}^{H}\text{T}(\hat{\theta })=[{\text{q}}_{1},\ldots ,{\text{q}}_{P-1},{\text{q}}_{P},{\text{q}}_{P+1},\ldots ,{\text{q}}_{2P-1}]$, where **q***_j_*, *j* = 1, …, 2*P* − 1 is the *j*-th column of **Q**(*θ̂*). By replacing the columns of **Q**(*θ̂*) and the associated elements of **v**(*θ̂*), Equation (16) can then be reformed as:

(17) $${\text{Q}}^{\prime }(\hat{\theta }){\text{v}}^{\prime }(\hat{\theta })=0$$

where **Q**′(*θ̂*) = [**q***_P_*, **q**_1_, …, **q***_P_*_−1_, **q***_P_*_+1_, …, **q**_2_*_P_*_−1_] and **v**′(*θ*) = [1, *μ*_1_, …, *μ_P_*_−1_, *α*_1_, …, *α_P_*_−1_]^T^. Moreover, extracting **q***_P_* from **Q**′(*θ̂*), Equation (17) can be expressed as:

(18) $${\text{Q}}^{\prime }(:,2:2P-1){\text{v}}^{\prime }(2:P)=-{\text{q}}_{P}$$

Consequently, we can get the estimates of *μ_k_* and *α_k_* by solving the above equation as:

(19) $${\text{v}}^{\prime }(2:P)=-{\text{Q}}^{\prime }{\left(:,2:2P-1\right)}^{\#}{\text{q}}_{P}$$

where (·)^#^ represents the pseudo inverse operation.

Now, we estimate the mutual coupling coefficients by utilizing the estimated ADCs *μ̂_k_*, *α̂_k_*. Based on the observation of *μ_k_*, *α_k_* in Equations (9) and (10), it is not difficult to find that they are the linear functions of *c_k_*, *k* = 1, …, *P* − 1. Let **B**(*θ̂*) be the coefficient matrix between **c**′ = [*c*_1_, …, *c_P_*_−1_]^T^ and [*μ̂*_1_,…, *μ̂_P_*_−1_, *α̂*_1_,…, *α̂_P_*_−1_]^T^. Then, we have:

(20) $$\text{B}(\hat{\theta }){\text{c}}^{\prime }={\text{v}}_{g}$$

where:

(21) $$\text{B}(\hat{\theta })=\left[\begin{array}{cccccc} \beta (\hat{\theta }) & \beta^{2}(\hat{\theta }) & \beta^{3}(\hat{\theta }) & \cdots & \beta^{P-2}(\hat{\theta }) & \beta^{P-1}(\hat{\theta })\\ \beta (\hat{\theta })+\beta^{-1}(\hat{\theta }) & \beta^{2}(\hat{\theta }) & \beta^{3}(\hat{\theta }) & \cdots & \beta^{P-2}(\hat{\theta }) & \beta^{P-1}(\hat{\theta })\\ \beta (\hat{\theta })+\beta^{-1}(\hat{\theta }) & \beta^{2}(\hat{\theta })+\beta^{-2}(\hat{\theta }) & \beta^{3}(\hat{\theta }) & \cdots & \beta^{P-2}(\hat{\theta }) & \beta^{P-1}(\hat{\theta })\\ \vdots & \vdots & \vdots & \ddots & \vdots & \vdots \\ \beta (\hat{\theta })+\beta^{-1}(\hat{\theta }) & \beta^{2}(\hat{\theta })+\beta^{-2}(\hat{\theta }) & \beta^{3}(\hat{\theta })+\beta^{-3}(\hat{\theta }) & \ldots & \beta^{P-2}(\hat{\theta })+\beta^{2-P}(\hat{\theta }) & \beta^{P-1}(\hat{\theta })\\ \beta (\hat{\theta })+\beta^{-1}(\hat{\theta }) & \beta^{2}(\hat{\theta })+\beta^{-2}(\hat{\theta }) & \beta^{3}(\hat{\theta })+\beta^{-3}(\hat{\theta }) & \ldots & \beta^{P-2}(\hat{\theta })+\beta^{2-P}(\hat{\theta }) & \beta^{1-P}(\hat{\theta })\\ \vdots & \vdots & \vdots & \ddots & \vdots & \vdots \\ \beta (\hat{\theta })+\beta^{-1}(\hat{\theta }) & \beta^{2}(\hat{\theta })+\beta^{-2}(\hat{\theta }) & \beta^{-3}(\hat{\theta }) & \cdots & \beta^{2-P}(\hat{\theta }) & \beta^{1-P}(\hat{\theta })\\ \beta (\hat{\theta })+\beta^{-1}(\hat{\theta }) & \beta^{-2}(\hat{\theta }) & \beta^{-3}(\hat{\theta }) & \cdots & \beta^{2-P}(\hat{\theta }) & \beta^{1-P}(\hat{\theta })\\ \beta^{-1}(\hat{\theta }) & \beta^{-2}(\hat{\theta }) & \beta^{-3}(\hat{\theta }) & \cdots & \beta^{2-P}(\hat{\theta }) & \beta^{1-P}(\hat{\theta }) \end{array}\right]$$

can be obtained easily if *θ̂* is estimated and:

(22) $${\text{v}}_{g}={\left[{\hat{\mu }}_{1}g_{0}-1,\ldots ,{\hat{\mu }}_{P-1}g_{0}-1,{\hat{\alpha }}_{1}g_{0}-1,\ldots ,{\hat{\alpha }}_{P-1}g_{0}-1\right]}^{\text{T}}$$

From Equations (20) and (22), we can see that *g*_0_ must be determined before the estimation of *C_k_*. Although containing the unknown mutual coefficient, *g_0_* can be easily obtained by employing the particular composition of *μ̂_k_*, *α̂_k_*. Notice that *μ̂*_1_ and *α̂_P_*_−1_ have the complementary elements of *g*_0_. We can therefore get *g*_0_ by:

(23) $${\hat{g}}_{0}=1/({\hat{\mu }}_{1}+{\hat{\alpha }}_{P-1})$$

where *μ̂*_1_ and *α̂_P_*_−1_ have been acquired from Equation (19).

Combining Equations (23) and (22), the mutual coupling coefficient vector **c**′ can be determined by solving Equation (20) as:

(24) $${\text{c}}^{\prime }={\text{B}}^{\#}(\hat{\theta }){\text{v}}_{g}$$

For better performance, all of the DOAs estimated in the above subsection can be used to form the extended coefficient matrix **B̃**. Let **B̃**. = [**B**^T^(*θ̂*_1_),…, **B**^T^(*θ̂_K_*)]^T^ and ${\tilde{\text{v}}}_{g}={\left[{\text{v}}_{g_{1}}^{\text{T}},\ldots ,{\text{v}}_{gK}^{\text{T}}\right]}^{\text{T}}$ with **B**(*θ̂_i_*) and v*_gi_* as the matrix and vector evaluated at the *i*-th estimated DOA *θ̂_i_*. Then, the extension of Equation (20) will be:

(25) $$\tilde{\text{B}}{\text{c}}^{\prime }={\tilde{\text{v}}}_{g}$$

Solving Equation (25) by the least squares, we can get a more precise estimation of **c**′ as:

(26) $${\text{c}}^{\prime }={\tilde{\text{B}}}^{\#}{\tilde{\text{v}}}_{g}$$

The above approach provides us a means of mutual coupling compensation. That is to say, once the vector **c**′ is determined, the matrix **C** can be formed by locating its element on the corresponding sub-diagonal. Therefore, DOA estimation can be further obtained by searching the peak of:

(27) $$P(\theta )=\frac{1}{{\left\|{\text{E}}_{n}^{\text{H}}\text{Ca}(\theta )\right\|}^{2}}$$

The performance can be further improved by repeating the above procedure. The proposed algorithm for DOA estimation and mutual coupling compensation can be summarized as follows.

(1) Get *L* snapshots of the received signal x(*t*) at *t* = *t*_1_, …, *t_L_*, and form the following matrix as:
  $$\text{X}=\left[\text{x}\left(t_{1}\right),\text{x}\left(t_{2}\right),\ldots ,\text{x}\left(t_{L}\right)\right]$$
(2) Generate the covariance matrix using the above data matrix by:
  $${\hat{\text{R}}}_{x}={\text{XX}}^{\text{H}}/L$$
(3) Conduct the EVD of **R̂***_x_*, and get the noise subspace **Ê***_n_*.
(4) Scan the direction from −90° to 90° with 1° as the step size. Calculate the special spectrum using Equation (14), and obtain the initial estimation of DOA *θ̂*_1_, …, *θ̂_K_*.
(5) For each *θ̂_i_*, estimate the ADCs *μ̂_k_*, *α̂_k_* for *k* = 1, …, *P* − 1 based on Equation (19). Then, calculate Equations (23) and (22) to obtain the values of *g*_0_ and v*_g_*, respectively.
(6) Form the matrix *B̃* and **v***˜_g_* by **B**(*θ̃_i_*) and **v***˜_gi_*, *i* = 1, …, *K*, and solve Equation (26) to get the parameters in the coupling matrix **C**.
(7) Enhance the DOA estimation with the estimated **C̃** and Equation (27).
(8) Repeat Step (5) to Step (7) to get a more precise estimation of directions.

## 4. Simulation Results

In this section, simulations will be conducted to validate the performance of the proposed method. Consider two independent sources from the far-field incident on the ULA from *θ*_1_ = −10° and *θ*_2_ = 20°. Sensors are located in the array with equal spacing *d* = *λ*/2. The number of effective mutual coupling coefficients is *P* = 3. Here, we set **c** = [1, 0.43301 − 0.25*i*, 0.14142 − 0.14142*i*], which is used in the second simulation of [17], to guarantee *g*_0_ ≠ 0 at any direction *θ*.

In the first simulation, we evaluate the performance of DOA estimation in Step (4) without mutual coupling compensation. Assume that the array number is *M* = 7 and that the snapshot number is 500. The root mean squared error (RMSE) is used to compare the DOA accuracy of different algorithms. It can be calculated, in general, by:

$$\text{RMSE}=\sqrt{\sum_{n=1}^{N_{S}}\sum_{i=1}^{K}{\left(\theta_{i}-{\hat{\theta }}_{i,n}\right)}^{2}/(KN_{S})}$$

where *θ̂_i_*_,_*_n_* is the *i*-th estimated direction obtained from the *n*-th Monte Carlo experiment, *K* = 2 is the number of sources and *N_s_* = 200 is the number of Monte Carlo experiments.

**Figure 1.**
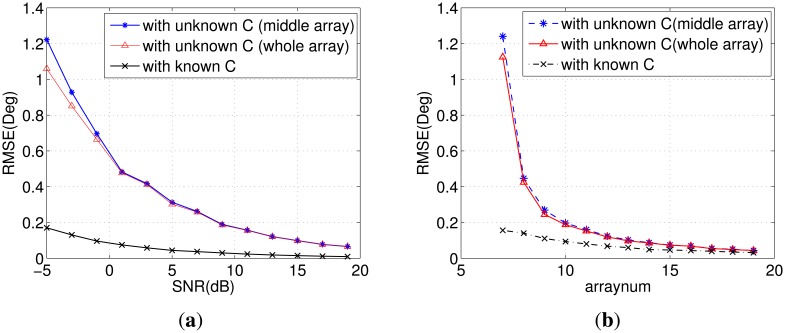
(**a**) RMSE of direction of arrival (DOA) *versus* SNR with a Monte Carlo experiment of 200 runs for array number *M* = 7 and snapshots *L* = 500; (**b**) RMSE of DOA *versus* the array number *M* with a Monte Carlo experiment of 200 runs for SNR = −5 dB and snapshots *L* = 500.

The RMSE as a function of SNR is illustrated in Figure 1a. The method used in our proposed algorithm is superior at low SNRs compared with the method in [17], since the whole array is utilized. As the SNR increases, the RMSE of DOA estimation decreases gradually for all of the methods. When the SNR is greater than 10 dB, the accuracy of the two methods with unknown **C** is almost the same. Figure 1b shows the effect of the array size on RMSE when SNR = −5 dB. Notice that the choice of *M* should satisfy Equation (15). It can be seen that our method slightly outperforms the method in [17] when *M* ≤ 10.

**Figure 2.**
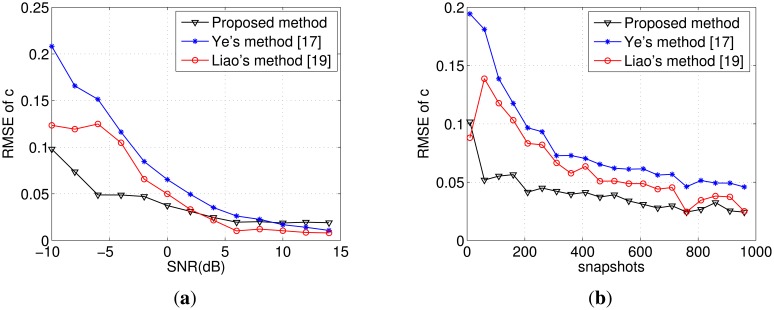
(**a**) RMSE of mutual coupling matrix (MCM) estimates *versus* SNR with a Monte Carlo experiment of 200 runs for array number *M* = *7* and snapshots *L* = 500; (**b**) RMSE of MCM estimates *versus* snapshots with a Monte Carlo experiment of 200 runs for array number *M* = 7 and SNR = −5 dB.

Using the initial DOA estimates, we now proceed to get the mutual coupling vector c. In the second simulation, we consider the same scenario. Define the RMSE of c as $\sqrt{\sum_{n=1}^{N_{S}}\sum_{k=1}^{P-1}{\left(c_{k}-{\hat{c}}_{k,n}\right)}^{2}/\left[N_{s}\left(P-1\right)\right]}$, where *Ĉ_k_*_,_*_n_* represents the estimated coupling coefficient in the *n*-th Monte Carlo experiment. From Figure 2a, we can see that the proposed method can obtain significant improvement of the coupling coefficient estimation when the SNR is lower than 3 dB. Besides, it is robust compared with the method in [17] and [19].

Now, we keep the SNR at −5 dB and vary the snapshots from 10 to 960. Figure 2b shows that the proposed method can achieve higher accuracy compared with the other two algorithms. From the estimation of the coupling matrix in Steps (5) and (6), it is not difficult to find that the full use of *μ̂_k_*, *α̂_k_* leads to the superior performance of the mutual coupling estimation.

**Figure 3.**
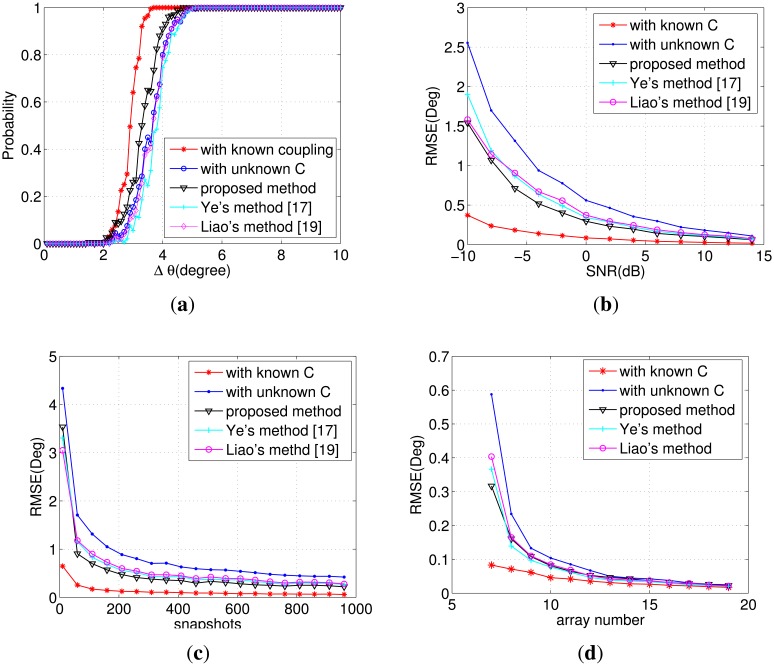
(**a**) Probability of DOA identification *versus* the angle interval for SNR = 0 dB, *M* = 7 and snapshots *L* = 500; (**b**) RMSE of DOA *versus* SNR with *M* = 7 and snapshots *L* = 500; (**c**) RMSE of DOA *versus* snapshots with *M* = 7 and SNR = 0 dB; (**d**) RMSE of DOA *versus* the array number with SNR = 0 dB and snapshots *L* = 500.

In the third simulation, we investigate the DOA estimation obtained by mutual coupling compensation. We consider two sources incoming from *θ*_1_ and *θ*_2_ = *θ*_1_ + Δ*θ* with SNR = 0 dB arriving at the ULA for *M* = 7 and *L* = 500. Define:

$$\left|\theta_{1}-{\hat{\theta }}_{1}\right|+\left|\theta_{2}-{\hat{\theta }}_{2}\right|<\left|{\hat{\theta }}_{2}-{\hat{\theta }}_{1}\right|$$

as the decision condition of whether the angles *θ̂*_1_, *θ̂*_2_ can be identified or not. Then, we conduct *Ns* = 200 Monte Carlo experiments for each algorithm and record the distinguishable ones. Denote *n_s_* as the number of experiments satisfying the above inequality. The curve of Probability = *n_s_/N_s_* with respect to the angle interval is presented in Figure 3a. Compared with the initial estimation of DOA, as shown in Step (4), the proposed method improves the accuracy dramatically by refinement with mutual coupling compensation. Ye's method [17] is inferior to the new method, because only the middle array is used. Figure 3b-d illustrates the RMSE of the DOA estimation with respect to the SNR, snapshots and array size, respectively. As expected, the results are very promising. It is the full use of x(*t*), *μ̂_k_* and *α̂_k_* that leads to the good performance of the proposed method. In the above simulations, the number of sensors is assumed to be seven. It is worth mentioning that similar results can be obtained for bigger *M*, as long as it satisfies *M* ≤ 10 for *P* = 3. The only difference is that the superiority is less obvious.

**Figure 4.**
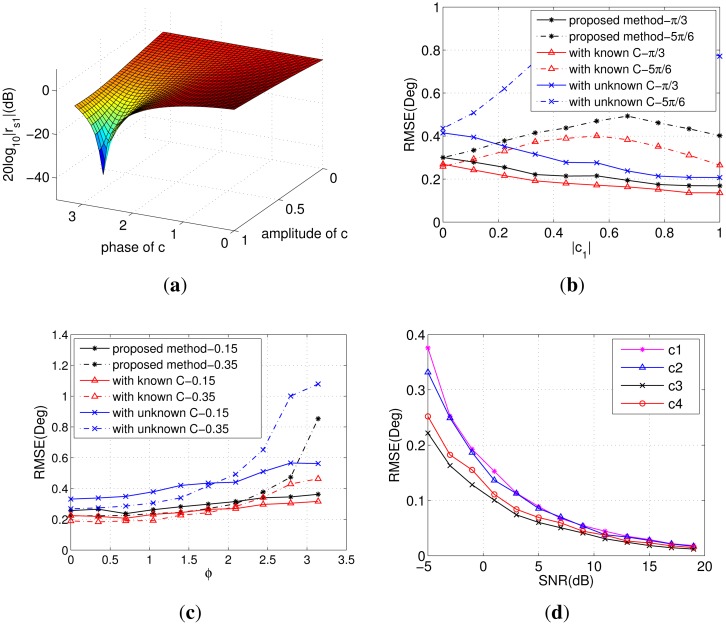
(**a**) The response of 1thelement with a single signal from −10° incident on the array; (**b**) RMSE of DOA *versus* |*c*_1_| with *ϕ* = *π*/3 and *ϕ* = 5*π*/6; (**c**) RMSE of DOA *versus ϕ* with |*c*_1_| = 0.15 and |c_2_| = 0.35; (**d**) RMSE of the proposed method *versus* SNR with different coupling coefficients.

In the fourth simulation, we will investigate the effect of mutual coupling on the DOA estimation. First, consider a signal impinging on the ULA from −10°. Mutual coupling between the adjacent two sensors exists, *i.e.*, *P* = 2. Then, define *r_si_* = **C**(*i*, :)**a**(*θ*),*i* = 1, …, *M* as the response of the *i*-th element to the received signal. *r_s_*_1_ is presented in Figure 4a with the amplitude and phase of the coupling coefficient varying. The figure illustrates that when the phase stays near zero, the response becomes higher as the amplitude increases. When the phase grows into the range of [*π*/2, π], the result will be the inverse to that of [0, *π*/2). The effect of coupling is to some extent similar to the beamforming. Any change of the amplitude and phase could make the response different.

The RMSE as a function of the amplitude of *c*_1_ is presented in Figure 4b with the phase *ϕ* fixed at *π*/3 and 5*π*/6, respectively. From this, we can see that the results get better with the increase of |*c*_1_| on the condition that *ϕ* = *π*/3. The situation gets worse for *ϕ* = 5*π*/6, since the response becomes gradually lower. Figure 4c demonstrates the RMSE as a function of the phase with |*c*_1_| fixed at 0.15 and 0.35, respectively. The error increases slightly as the phase grows from zero to *π*, which coincides with the response shown in Figure 4a. Figure 4d presents the RMSE of the proposed method *versus* SNR with different coupling coefficients. In this experiment, we assume that there are two signals from −10° and 20° impinging on the ULA and set the four coefficients as **c**_1_ = [1, −0.2801 − 0.254*i*, −0.14 − 0.14*i*], **c**_2_ = [1, −0.125 + 0.108*i*, −0.066 + 0.858*i*], **c**_3_ = [1, 0.2801 + 0.254*i*, 0.14 + 0.14*i*] and **c**_4_ = [1, 0.125 + 0.108*i*, 0.066 + 0.858*i*]. From Figure 4d we can conclude that the proposed algorithm could achieve better performance when the coupling coefficient has a smaller phase and greater amplitude.

**Figure 5.**
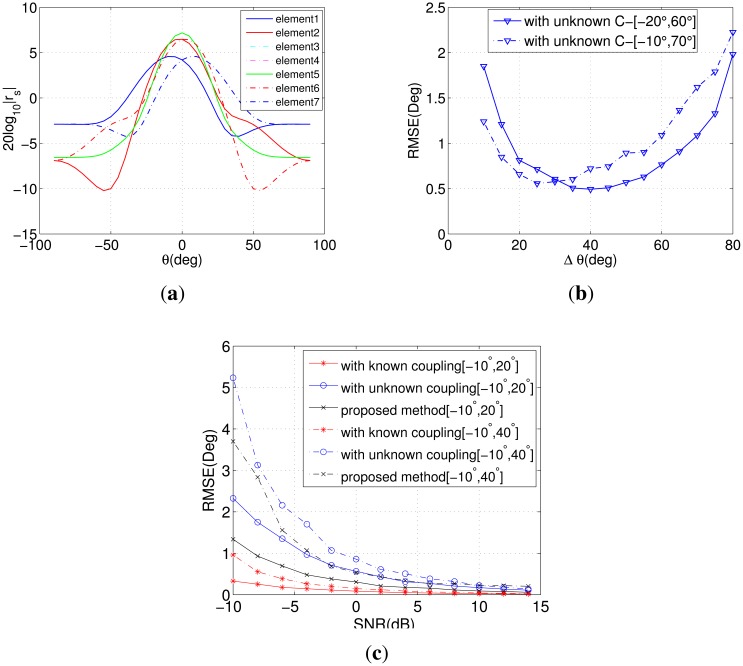
(**a**) The response of different elements *versus* DOA; (**b**) RMSE of DOA estimation *versus* the angle interval; (**c**) RMSE of DOA estimation *versus* SNR with different angle intervals.

In the fifth simulation, we access the performance of DOA estimation when signals impinge on the ULA with different angle intervals. Figure 5a presents the response of every element to DOA with **c** = [1, 0.43301 − 0.25*i*, 0.14142 − 0.14142*i*]. Figure 5b illustrates the influence of the angle interval to the initial estimation in Step (4). It is shown that not only the angle interval, but also the initial DOA will affect the accuracy of estimation seriously. As long as the DOAs locate in the main lobe of the middle element, the gain of the array will not decline rapidly. Figure 5c gives the RMSE of DOA estimation *versus* SNR with different angle intervals. With the same initial DOA, the performance obviously decreases when the interval becomes bigger.

In summary, the proposed method can achieve more precise estimates of DOA as opposed to Ye's method [17] and Liao's method [19], especially when the SNR is low and the array size is close to the minimum available value of *M* = K+2*P*−1. The reservation of the information obtained guarantees the good performance of the proposed algorithm. The experiments of the mutual coupling effect show that the coupling coefficient, initial DOA and the angle interval could have an influence on the performance of DOA estimation at the same time.

## 5. Conclusions

This paper addresses the DOA estimation in the presence of unknown mutual coupling. Based on the subspace theory, the initial DOA is first estimated using the whole array without calibration sources and auxiliary sensors, which leads to high accuracy. With the assumption that no blind angles exist in the space, mutual coupling compensation is further conducted by estimating the coupling coefficients indirectly from the angular-dependent coefficients. Finally, with all of the ADCs utilized without discarding any, the mutual coupling coefficients are determined by solving the least squares problem. Simulations show that the proposed method can achieve better performance at low SNR with a small-sized array. The robustness of the method can be verified, as well.
